# A Resonant Piezoelectric Diaphragm Pump Transferring Gas with Compact Structure

**DOI:** 10.3390/mi7120219

**Published:** 2016-12-01

**Authors:** Jiantao Wang, Yong Liu, Yanhu Shen, Song Chen, Zhigang Yang

**Affiliations:** School of Mechanical Science and Engineering, College of Biological and Agricultural Engineering, Jilin University, Changchun 130025, China; jiantao.888@163.com (J.W.); yongliu@jlu.edu.cn (Y.L.); chensong13@mails.jlu.edu.cn (S.C.); yzg@jlu.edu.cn (Z.Y.)

**Keywords:** rectangular piezoelectric vibrator, resonance, piezoelectric diaphragm pump, piezoelectric gas pump, diaphragm system

## Abstract

In order to improve the output capacity of a piezoelectric pump when transferring gas, this paper presents a compact resonant piezoelectric diaphragm pump (hereinafter referred to as the piezoelectric diaphragm pump), which is driven by a rectangular piezoelectric vibrator. The compact structure can effectively release the vibrating constraints of the vibrator, and enlarge its center output displacement, so as to increase the volume change rate of the pump chamber. Based on the structure and the working principle of this piezoelectric diaphragm pump, a dynamic model for the diaphragm system is established in this paper, and an analysis on factors affecting the resonant frequency of the system is then conducted. We tested on the prototype under the driving voltage of 260 Vpp. The results show that the diaphragm system reaches resonance under the driving frequency of 265 Hz, which is very close to the fundamental frequency of check valve. Compared with the rectangular piezoelectric vibrator’s amplitude, the diaphragm’s amplitude is double amplified. At this time, the piezoelectric diaphragm pump achieves the maximum gas flow rate as 186.8 mL/min and the maximum output pressure as 56.7 kPa.

## 1. Introduction

Decades of development has given the piezoelectric diaphragm pump an extensive application and a favorable prospect. The media it can transfer include micro-compressible liquid with low viscosity, high viscosity liquid, particle flow, and compressible gas [1,2,3]. Commonly, the diaphragm of a piezoelectric diaphragm pump is the piezoelectric vibrator itself. However, it has a low transfer capacity when conveying compressible gas, constrained by the small deformation of this kind of vibrator [4]. To solve this problem, researchers separated the pump’s diaphragm and the piezoelectric vibrator and amplified the vibrator’s deformation (by using the principle of resonance) to drive the diaphragm, so that the transfer capacity of the piezoelectric diaphragm pump can be improved [5].

The document regarding the resonant piezoelectric pump is first seen in June-Ho Park’s study [6,7,8]. The pump is constituted by the piezoelectric stack, added mass, bellows, and check valve. With water as the transfer medium, the maximum output flow rate is 4.8 mL/min, and the maximum pumping pressure is 0.32 MPa, but the output capacity of gas is unknown. In 2007, the Lyndon Johnson Space Center in Texas successfully developed a resonant piezoelectric diaphragm pump, of which the diaphragm movement in the pump chamber was driven by a piezoelectric stack [9]. In a resonance state, the displacement of the piezoelectric stack is amplified 50 times, greatly improving the flow rate of the piezoelectric diaphragm pump. However, as the piezoelectric stack have complex manufacturing process and high cost, this resonant piezoelectric diaphragm pump was failed in large-scale applications.

For the precise conveying of fuel cells, in 2014, the University of Science and Technology of China presented a resonant piezoelectric diaphragm pump, which has compressible spaces at the inlet and outlet of the pump chamber [10,11]. The external dimensions of the pump are 100 mm × 20 mm × 15 mm. It is driven by the vibrating inertia of the piezoelectric vibrator. When the drive voltage is 400 V and the driving frequency is 490 Hz, the maximum liquid flow rate is 105 mL/min, and the maximum pumping pressure is 23 kPa.

The number of the studies on gas piezoelectric diaphragm pump is not large. In 2013, Jilin University in China successfully developed a new type of resonant gas piezoelectric diaphragm pump, which adopts a low-cost annular piezoelectric bimorph as the driving source, constructing the resonance system [12]. It enlarged the displacement of the piezoelectric vibrator 4.2 times, with a gas flow rate reaching 1685 mL/min. However, this diaphragm pump has such disadvantages as a complicated structure, a large volume, and significant working noise.

In order to improve the gas output capacity and achieve a compact structure, a resonant piezoelectric diaphragm pump that is driven by a rectangular bimorph is presented in this paper. The two fixed ends of the rectangular piezoelectric vibrator can improve the stability of the diaphragm vibration and reduce the impact that the installation method has on the pump’s output performance. The vibrator and the diaphragm are connected by an elastic mass. By changing its mass and stiffness, the resonant frequency of the system can be adjusted conveniently. Moreover, the relatively open structure facilitates the heat radiating of the piezoelectric vibrator.

In this paper, we conduct a dynamic analysis on the diaphragm system of the piezoelectric diaphragm gas pump and identify the factors that affect the diaphragm amplitude. We designed and produced a prototype of resonant piezoelectric diaphragm gas pump, and we then tested and analyzed its gas output performance. By adjusting the parameters of the diaphragm vibration system, the performance of the pump has been optimized to the maximum.

## 2. Structure and Working Principle

### 2.1. Structure

**Figure 1:**
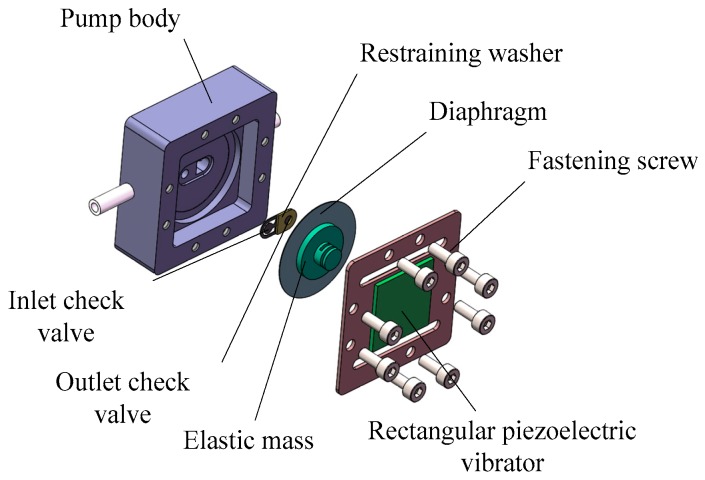
Structure of the piezoelectric diaphragm gas pump.

The structure of the compact resonant piezoelectric diaphragm pump developed in this study is shown in Figure 1. It consists of a pump body, a rectangular piezoelectric vibrator, elastic mass, a diaphragm, an inlet check valve, an outlet check valve, and a restraining washer. Specifically, the rectangular piezoelectric vibrator, the driving element, is a rectangular metal substrate with a rectangular bimorph adhered to both its sides. The elastic mass does not only transmit the vibration for the system, but also adjusts the system’s resonance frequency by changing its stiffness and mass. The check valve, which employs a cantilever structure, is processed by UV laser cutting, with PET (polyethylene terephthalate) as the material. The diaphragm is made from a circular beryllium bronze sheet, of which the circumferential edge is bonded and sealed to the pump chamber via epoxy.

### 2.2. Working Principle

**Figure 2:**
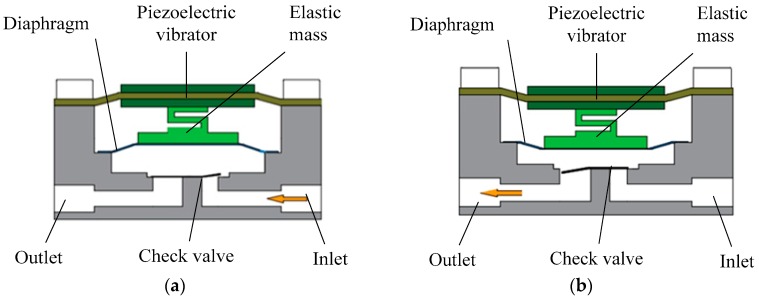
Working principle of piezoelectric diaphragm pump. (**a**) Suction process; (**b**) Discharge process.

When the AC (alternating current) voltage is applied to the rectangular piezoelectric vibrator, it will have a periodically reciprocating vibration, but the amplitude is small. When the excitation frequency reaches the resonant frequency of the system, the diaphragm system, which includes the rectangular vibrator, elastic mass and the diaphragm, will achieve resonance. Consequently, the amplitude of the rectangular piezoelectric vibrator will be amplified. The diaphragm vibrating leads to periodic changes of the pump chamber volume. Under the coordination of the two check valves, the process of the fluid suction and discharge is formed. The constant suction and discharge eventually achieve the fluid’s one-way flow, as shown in Figure 2.

## 3. Dynamic Model

**Figure 3:**
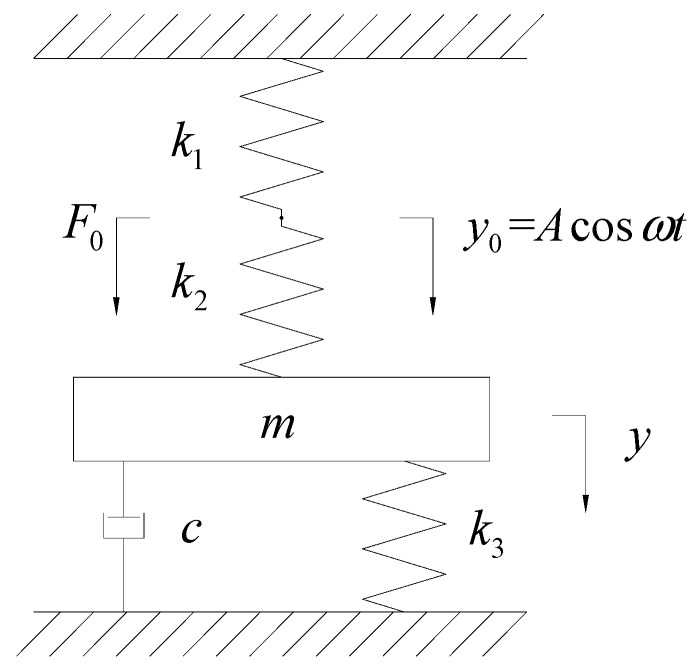
Dynamic model of piezoelectric diaphragm pump.

The interaction among gas, the diaphragm, and the check valves is complicated. Therefore, in order to simplify the analysis objects, the diaphragm system composed by the rectangular piezoelectric vibrator, the elastic mass, and the diaphragm is considered as an independent vibration system. Among them, the vibrator is considered as a massless ideal spring that provides vibrating excitation, and the elastic mass and the diaphragm are regarded as the perfect elastic and mass components, ignoring their vibration damping. The interaction of gas, the flow passage, and the check valve is equivalent to damping *c*. The dynamic model of the piezoelectric diaphragm gas pump is shown in Figure 3. Wherein m is the equivalent mass of the elastic mass and the diaphragm, $k_{1}$ is the equivalent stiffness of the rectangular piezoelectric vibrator, $k_{2}$ is the stiffness of the elastic mass, and $k_{3}$ is the stiffness of the diaphragm.

Assuming the driving frequency of the piezoelectric vibrator is ω, the vibration amplitude is A, then the vibration displacement of the rectangular piezoelectric vibrator is $y_{0}=A\cos\omega t$. Assuming the displacement of the diaphragm is y, the motion differential equation of the system is

(1) $$\left\{ \begin{array}{l} m\ddot{y}+c\dot{y}+k_{2}\left(y-y_{0}\right)+k_{3}y=0\\ F_{0}+k_{2}\left(y-y_{0}\right)-k_{1}y_{0}=0 \end{array}\right..$$

Obtained by Equation (1),

(2) $$m\ddot{y}+c\dot{y}+\frac{k_{1}k_{2}+k_{1}k_{3}+k_{2}k_{3}}{k_{1}+k_{2}}y=\frac{k_{2}F_{0}}{k_{1}+k_{2}}.$$

Obtained by Equation (2),

(3) $$\ddot{y}+\frac{c}{m}\dot{y}+\frac{k_{1}k_{2}+k_{1}k_{3}+k_{2}k_{3}}{m\left(k_{1}+k_{2}\right)}y=\frac{k_{2}F_{0}}{m\left(k_{1}+k_{2}\right)}.$$

According to Equation (3), the natural frequency of the system is $\omega_{n}=\sqrt{\frac{k_{1}k_{2}+k_{1}k_{3}+k_{2}k_{3}}{m\left(k_{1}+k_{2}\right)}}$, and the damping ratio is $\zeta =\frac{c}{2m\omega_{n}}$; therefore, we obtain Equation (4),

(4) $$\ddot{y}+2\zeta \omega_{n}\dot{y}+\omega_{n}^{2}y=\frac{k_{1}k_{2}}{k_{1}k_{2}+k_{1}k_{3}+k_{2}k_{3}}A\omega_{n}^{2}\cos\omega t.$$

To calculate and obtain the steady-state response of the system, we use the following Equation (5):

(5) $$y=\frac{k_{1}k_{2}}{k_{1}k_{2}+k_{1}k_{3}+k_{2}k_{3}}•\frac{A\cos\omega t}{{\left[1-{\left(\frac{\omega }{\omega_{n}}\right)}^{2}\right]}^{2}+{\left(2\zeta \frac{\omega }{\omega_{n}}\right)}^{2}}.$$

The amplitude amplification coefficient of vibration system is

(6) $$\left|H\left(\omega \right)\right|=\frac{Y}{A}=\frac{k_{1}k_{2}}{\left(k_{1}k_{2}+k_{1}k_{3}+k_{2}k_{3}\right)\sqrt{{\left[1-{\left(\frac{\omega }{\omega_{n}}\right)}^{2}\right]}^{2}+{\left(2\zeta \frac{\omega }{\omega_{n}}\right)}^{2}}}.$$

When the driving frequency $\omega \;=\;\omega_{n}$, the system reaches a resonance state; at this time, based on Equation (6), the amplitude amplification coefficient of the vibration system reaches the maximum, as shown by Equation (7):

(7) $$\left|H\left(\omega \right)\right|=\frac{k_{1}k_{2}}{2\zeta \left(k_{1}k_{2}+k_{1}k_{3}+k_{2}k_{3}\right)}.$$

When the driving frequency is equal to the resonant frequency of the piezoelectric diaphragm gas pump, the volume change rate of the pump chamber can be increased, so as to improve the capacity of conveying gas. Accordingly, when the external driving frequency is a given value, it is very significant that the resonance frequency of the diaphragm system can be adjusted to approach the value. According to Equation (3), we can change the resonant frequency of the system effectively by changing the mass and stiffness of the elastic mass.

## 4. Performance Testing and Structure Optimization

When the piezoelectric pump is driven by the piezoelectric vibrator directly, its optimal working frequency is mainly determined by the resonant frequency of the check valve, regardless of conveying gas or liquid [13]. In addition, when the equivalent stiffness of the check valve is small, the fundamental frequency is low. At this time, the opening degree of the check valve is large, resulting in a small flow resistance, which entails the enhancement of the output performance of the pump. Based on the above principle, we employed a PET film, whose elastic modulus is small, as the material of the check valve in this study.

In the testing stage, we measured the fundamental frequency of the given check valve at first. Based on the analyzing results of the dynamic model in Section 3, we changed the mass of the elastic mass block to make the resonant frequency of the diaphragm system as close to the fundamental frequency of the check valve as possible, so as to optimize the output performance of the piezoelectric diaphragm pump.

### 4.1. Prototype Parameters

**Figure 4:**
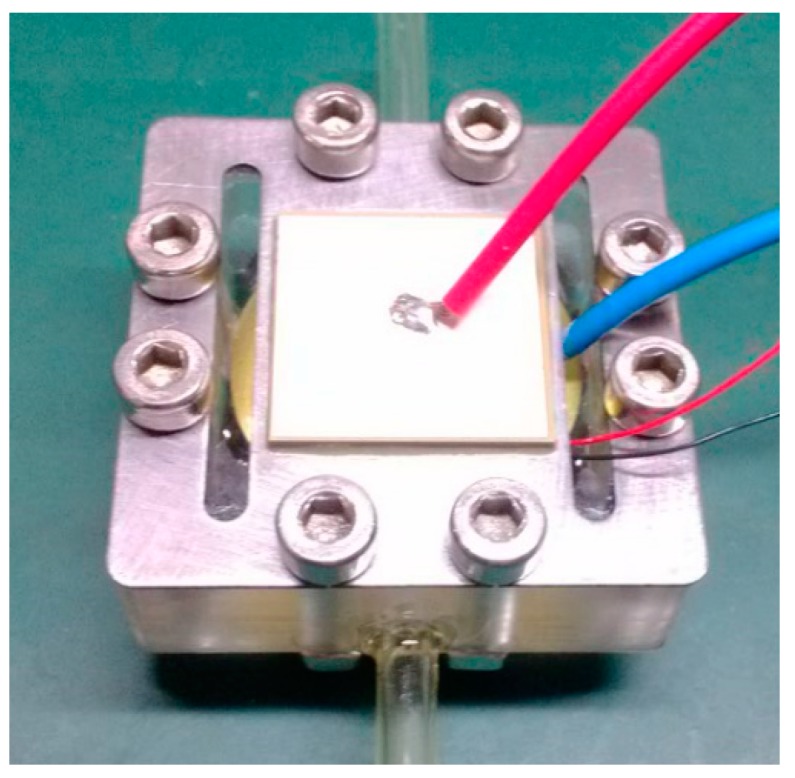
Dynamic model of piezoelectric diaphragm pump.

Figure 4 shows a prototype photo of the piezoelectric diaphragm pump presented in this paper. Its external dimensions are 52 mm × 52 mm × 36 mm. The piezoelectric bimorph is square with a side length of 20 mm; the metal substrate of the vibrator is 52 mm × 21 mm × 0.5 mm. The diaphragm is made of beryllium bronze, with a diameter of 21 mm and a thickness of 0.2 mm. The mass of the elastic mass block is 10 g, and the equivalent stiffness is 346 N/mm. The check valve is made of PET, with a thickness of 0.03 mm. The pump body is made from PMMA (polymethyl methacrylate), a highly transparent material, so that we can observe the check valve’s working state inside the pump chamber during the test. The diameter of the pump inlet and the outlet channel is 2.5 mm.

**Figure 5:**
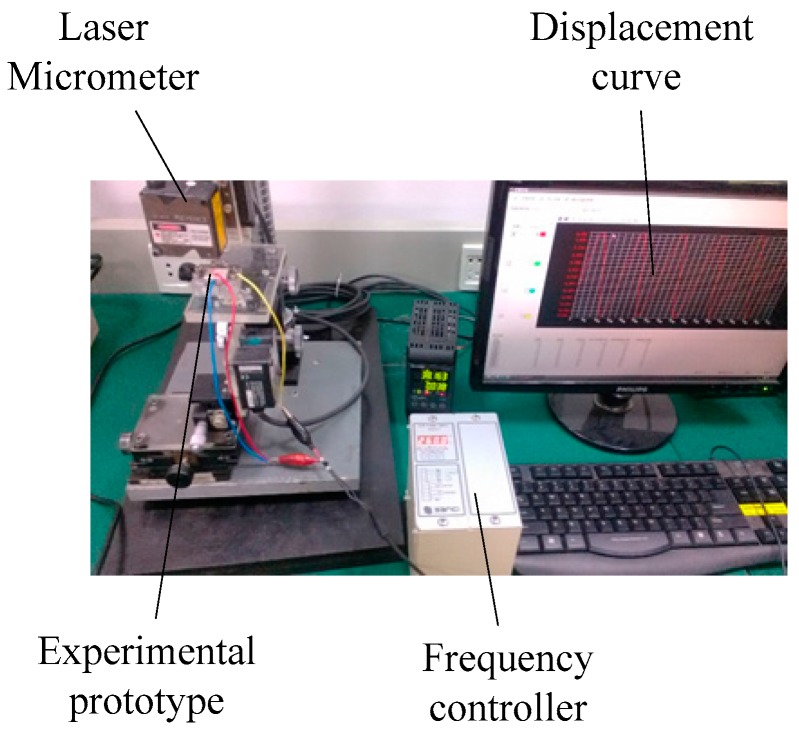
Performance test of the piezoelectric diaphragm gas pump.

The main equipment used during the test consists of a numeric piezoelectric frequency controller, a precision impedance analyzer, a laser micrometer, and a digital pressure gauge. During the test, the inlet pipe communicated with the atmosphere. We used the drainage method to measure the flow rate of the piezoelectric diaphragm pump. The performance test of the piezoelectric diaphragm gas pump is shown in Figure 5, when the driving signal is a sine wave with 260 Vpp.

### 4.2. Prototype’s Optimum Working Frequency Test

We applied an initial vibration excitation to the check valve installed in the prototype and used the laser micrometer to test the vibration curve of the check valve, achieving the fundamental frequency of 292 Hz for the check valve.

**Figure 6:**
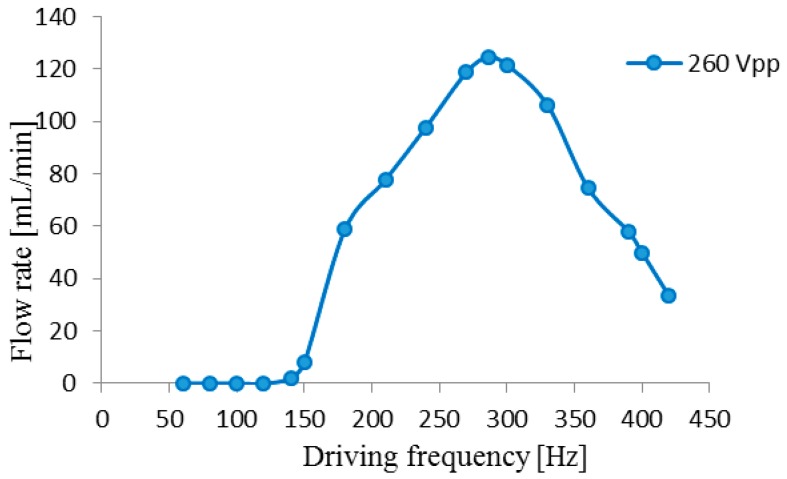
Flow rate—driving frequency curve.

The driving signal of the rectangular piezoelectric vibrator is a sine wave. When the driving voltage was maintained 260 Vpp, we changed the drive frequency constantly and obtained the flow rate–driving frequency curve, shown in Figure 6. From the figure, it is evident that, when the drive frequency was 286 Hz, the output flow rate of the piezoelectric diaphragm gas pump reached the maximum of 124.6 mL/min. Therefore, we can conclude that the optimal frequency of the prototype is 286 Hz, which is nearly equal to the fundamental frequency of the check valve.

### 4.3. Performance Optimization

Based on the analysis and calculation on the dynamic model of the diaphragm system, we adjusted the elastic mass of the piezoelectric pump prototype to 10 g, 30 g, 60 g, 80 g, and 100 g, respectively, and tested the resonant frequency of the diaphragm system using the precision impedance analyzer. Results are shown in Table 1.

**Figure 7:**
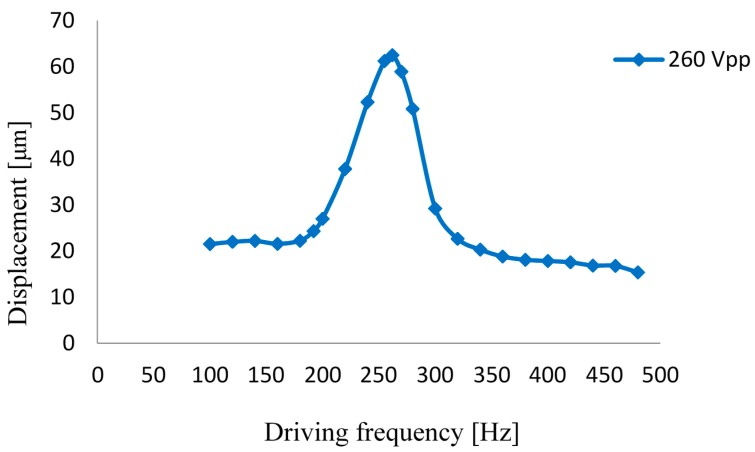
Diaphragm amplitude—frequency curve.

Only when the mass of the elastic mass block is 80 g, the resonant frequency of the system is close to the fundamental frequency of the check valve. Based on this, we tested the output performance of this prototype. The diaphragm amplitude–frequency characteristic of the prototype is shown in Figure 7 for a drive voltage of 260 Vpp. The curve indicates that, when the driving frequency was 265 Hz, we achieved the maximum amplitude of 62.5 μm for the diaphragm, and the maximum amplitude of the rectangular piezoelectric vibrator was 30 μm. It can be seen when the system is resonant, the displacement of the rectangular piezoelectric vibrator is amplified twice.

**Figure 8:**
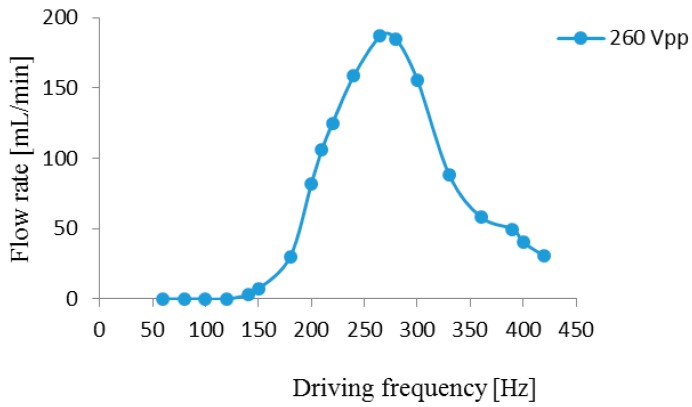
Flow rate—driving frequency curve.

**Figure 9:**
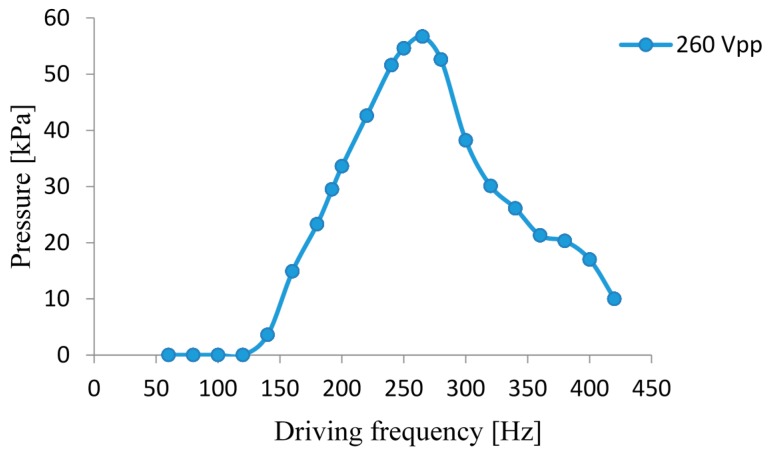
Output pressure—driving frequency curve.

Test results of the gas flow rate and the gas output pressure of the piezoelectric diaphragm pump prototype are illustrated in Figure 8 and Figure 9, respectively, for a drive voltage of 260 Vpp. The two graphs show an identical tendency. When the drive frequency is 265 Hz, the piezoelectric diaphragm gas pump achieves the maximum gas flow rate as 186.8 mL/min and the maximum output pressure as 56.7 kPa.

## 5. Conclusions

This paper presents a resonant piezoelectric diaphragm gas pump driven by a rectangular piezoelectric vibrator. It constructs a vibration system with two degrees of freedom. Under the given structure, tests on the pump demonstrate that, when the elastic mass is 80 g, the equivalent stiffness is 346 N/mm. The impedance analyzer shows the resonant frequency of the diaphragm system is 269 Hz, which is close to the fundamental frequency of the check valve. After the performance testing, we know the optimum working frequency of the pump is 265 Hz. At this time, the vibrating displacement of the piezoelectric vibrator is double amplified; the pump achieves the maximum gas flow rate as 186.8 mL/min and the maximum output pressure as 56.7 kPa.

It should be noted that this conclusion has some limitations. In general cases, when the fundamental frequency of the check valve is low, its opening degree is large, so the small flow resistance helps to improve the output performance of the piezoelectric pump in a single work cycle. This experiment demonstrates, if we adjust the resonant frequency of the diaphragm system to make it close to the fundamental frequency of the check valve, we can further improve the gas output performance of the pump effectively. However, the above method has a marked effect on the gas output pressure, but not necessarily leads to the maximum flow rate. The output flow rate of the pump is determined by both the external driving voltage and the flow rate of a single work cycle. Their complicated relationship is not fully discussed in this paper, which could provide other researchers with implications for further research.

## Figures and Tables

**Table 1:** Resonant frequency of the diaphragm system.

**Elastic Mass (g)**	10	30	60	80	100
**Resonant Frequency (Hz)**	741	427	302	269	117

## References

[1] Lee S.-M., Kuan Y.-D., Sung M.-F. (2013). Diaphragm air-liquid micro pump applicable to the direct methanol fuel cell. J. Power Sources.

[2] Ma H.-K., Hou B.-R., Wu H.-Y., Lin C.-Y., Gao J.-J., Kou M.-C. (2008). Development and application of a diaphragm micro-pump with piezoelectric device. Microsyst. Technol..

[3] Wen J.-M., Cheng G.-M., Kan J.-W. (2010). Study on value body of piezoelectric pump with active valve. J. Drain. Irrig. Mach. Eng..

[4] Senousy M.S., Li F.X., Mumford D. (2009). Thermo-electro-mechanical performance of piezoelectric stack actuators for fuel injector applications. J. Intell. Mater. Syst. Struct..

[5] Kan J.-W., Tang K.-H., Shao C.-H., Zhu G.-R. (2010). Performance analysis of a piezohydraulic motor. J. Harbin Eng. Univ..

[6] Park J.-H., Yoshida K., Yokota S. (1999). Resonantly driven piezoelectric micropump fabrication of a micropump having high power density. Mechatronics.

[7] Park J.-H., Yoshida K., Nakasu Y., Yokota S. A resonantly-driven piezoelectric micropump for microfactory. Proceedings of the 6th International Conference on Mechatronics Technology.

[8] Park J.-H., Yoshida K., Yokota S., Seto T., Takagi K. Development of micro machines using improved resonantly-driven piezoelectric micropumps. Proceedings of the 4th International Symposium on Fluid Power Transmission and Control.

[9] Lyndon B. Diaphragm Pump with Resonant Piezoelectric Drive. http://www.techbriefs.com/component/content/article/2182.

[10] Wang X.-Y., Ma Y.-T., Yan G.-Y., Huang D., Feng Z.-H. (2014). High flow-rate piezoelectric micropump with two fixed ends polydimethylsiloxane valves and compressible spaces. Sens. Actuators A Phys..

[11] Wang X.-Y., Ma Y.-T., Yan G.-Y., Feng Z.-H. (2014). A compact and high flow-rate piezoelectric micropump with a folded vibrator. Smart Mater. Struct..

[12] Wu Y., Liu Y., Liu J.-F., Wang L., Jiao X.-Y., Yang Z.-G. (2012). An improved resonantly driven piezoelectric gas pump. J. Mech. Sci. Technol..

[13] Liu Y. (2012). Theoretical & Experimental Study on Wheel Valve Micro-Piezoelectric Pump. Ph.D. Thesis.

